# Calcium Availability Influences Litter Size and Sex Ratio in White-Footed Mice (*Peromyscus leucopus*)

**DOI:** 10.1371/journal.pone.0041402

**Published:** 2012-08-01

**Authors:** Christina M. Schmidt, Wendy R. Hood

**Affiliations:** Department of Biological Sciences, Auburn University, Auburn Alabama, United States of America; Pennsylvania State University, United States of America

## Abstract

The production of offspring typically requires investment of resources derived from both the environment and maternal somatic reserves. As such, the availability of either of these types of resources has the potential to limit the degree to which resources are allocated to reproduction. Theory and empirical studies have argued that mothers modify reproductive performance relative to exogenous resource availability and maternal condition by adjusting size, number or sex of offspring produced. These relationships have classically been defined relative to availability of energy sources; however, in vertebrates, calcium also plays a critical role in offspring production, as a considerable amount of calcium is required to support the development of offspring skeleton(s). We tested whether the availability of calcium influences reproductive output by providing female white-footed mice with a low-calcium or standard diet from reproductive maturity to senescence. We then compared maternal skeletal condition and reproductive output, based on offspring mass, offspring number and litter sex ratio, between dietary treatments. Mothers on the low-calcium diet exhibited diminished skeletal condition at senescence and produced smaller and strongly female-biased litters. We show that skeletal condition and calcium intake can influence sex ratio and reproductive output following general theoretical models of resource partitioning during reproduction.

## Introduction

Life history theory predicts that mothers should optimize their reproductive success by partitioning resources in a manner that will afford them greatest fitness [1], [2]. To this end, maternal attributes (e.g. condition, age or parity), and the availability of exogenous resources may interact to influence the degree to which resources are invested into the production of offspring. Specifically, the number, size and, under certain conditions, sex of offspring produced at a given time may be affected by these factors.

Interactions between endogenous nutrient reserves and the availability of nutrients in an animal’s environment serve to establish the resource pool which ultimately limits the quantity of nutrients that can be devoted to the production of offspring (but see [3]). For example, poor body condition and food scarcity experienced by mothers during severe El Niño events is associated with reduced offspring body condition in California sea lions (*Zalophus californianus*) [4], and maternal body condition (residuals of body mass/skeletal size) is positively correlated with weaning mass under typical conditions in Columbian and Richardson’s ground squirrels (*Urocitellus columbianus, U. richardsonii*) [5], [6]. Greater maternal body fat stores and greater body fat utilization by mothers in polar bears (*Ursus maritimus*) and moose (*Alces alces*) has also been correlated with greater weaning mass [7], [8]. And, the availability of food is positively correlated with litter size in Prairie voles (*Microtus ochrogaster*) and Columbian ground squirrels [9], [10].

Trivers and Willard [11] proposed that for polygynous species in which the reproductive success of male offspring is more condition-dependent than that of female offspring, mothers in good condition should produce more male progeny than mothers in poor condition. In both roe deer (*Capreolus capreolus*) and reindeer (*Rangifer tarandus*), greater maternal body mass has been associated with the production of a higher proportion of male offspring [12], [13]; however, support for the Trivers-Willard hypothesis in mammals, including subsequent studies in the aforementioned species, is generally equivocal [14], [15], [16], [17], and has only been consistently demonstrated when maternal condition is quantified around the time of conception [18], [19].

The effect of maternal condition on reproductive output has principally been examined relative to the availability of energy sources (see [3], [14], [15], [20] for reviews). Individual differences in maternal condition are frequently quantified based on direct or indirect estimates of endogenous stores of fat, representing the body’s primary energy reserve. However, condition is best described as a measure of the functionality of cellular processes in the body, which encompasses a broad array of genetic and phenotypic components [21] that are not necessarily be associated with energy utilization. Regarding resources required for the production of young, a substantial amount of calcium must also be partitioned to mammalian offspring to support skeletal development and mineralization. Available calcium is traded-off between maternal skeletal stores and developing bone in their young. Thus, processes supporting maternal and offspring skeletal growth and integrity must also be considered to be vital components of condition [22], [23].

Maternal bone loss during gestation and lactation has been observed in several species of mammals [24], [25], [26]. Although bone loss increases the risk of sustaining a fracture [27], [28] and thus can be assumed to decrease a mother’s probability of survival, mothers commonly maintain mineral allocation to their young by supplementing ingested calcium with calcium from their own skeleton [22], [23], [29], [30], [31], [32], [33], [34]. As such, a trade-off likely exists between maternal skeletal condition and offspring production in mammals. Small mammals may be more susceptible to this interaction for three reasons. First, food, and thus nutrient intake, in small species may be limited by gastrointestinal capacity to absorb nutrients or the body’s ability to partition available nutrients to offspring [35]. Second, small mammals possess proportionately smaller skeletons, and thus relatively smaller calcium reserves, than larger mammals [36], [37]. Finally, small mammals transfer relatively large amounts of calcium to their offspring, based on high milk outputs [38] and milk calcium concentrations that are comparable to other mammals [39].

Few studies have addressed how calcium intake affects reproductive output in mammals outside of addressing offspring skeletal characteristics. It has been shown that big brown bats (*Eptesicus fuscus*) produce larger offspring relative to maternal mass when fed a calcium supplement [40], and hypocalcemia has been associated with diminished litter size and female fertility in rats [14], [41]. With regard to sex ratio, female rodents tend to produce fewer male offspring when stressed by nutritional deficiencies [14], however the potential impact of calcium deficiency on sex ratio has not been previously investigated.

Given the cost incurred by the maternal skeleton associated with offspring production, and that skeletal condition reflects both exogenous and endogenous calcium availability, we hypothesized that mammals adjust reproductive output relative to exogenous calcium availability and endogenous calcium stores in the skeleton. Specifically, we predicted that females with low calcium intake and lower bone mineral content, as indicated by the architectural characteristics of bone at key sites of mobilization, would give birth to fewer or smaller young, and that the primary sex ratio of the young would be skewed toward females. To test this, we manipulated calcium intake of captive white-footed mice (*Peromyscus leucopus*) to assess the relationship between dietary calcium, maternal skeletal condition, and parameters of reproductive performance over an individual’s lifetime. We also considered the effects of maternal age, parity, body mass, and calcium intake on factors associated with reproductive performance. White-footed mice lose bone over the course of gestation and lactation, and bone loss is intensified when mothers are consuming a low-calcium diet [42]. White-footed mice also allocate less calcium to individual offspring when dietary intake is reduced [42], thus making them a good model for testing the Trivers-Willard hypothesis within the context of calcium utilization.

## Materials and Methods

### Ethics Statement

All procedures followed recommendations in the Guide for the Care and Use of Laboratory Animals of the National Institutes of Health. This protocol was approved by the Auburn University Institutional Animal Care and Use Committee (protocol number: 2008–1365).

We obtained 15 nulliparous female and 8 male white-footed mice from the Peromyscus Genetic Stock Center (University of South Carolina, Columbia, SC USA) and maintained them at an animal facility at Auburn University (Auburn, AL USA) for the duration of the study. Females were housed in pairs in 29×19×12.5 cm polypropylene rodent boxes (Lab Products, Inc., Seaford, DE USA) at 25°C on a 16∶8 h light cycle. We provided females with *ad-lib* water and access to one of two custom manufactured diets that differed only in calcium content (modified TD.08174, Harlan Teklad, Madison, WI USA; Table 1). Seven females received a low-calcium diet (0.10% calcium) and 8 females received a standard diet (0.85% calcium), which provided recommended calcium intake for reproductive mice (Harlan Teklad, pers. comm.) We provided females with 4–5 breeding opportunities over a period of 75 weeks. We used a random number generator to select males for pairing with females and females were never paired with the same male more than once. Males were comparable in age to the females and were maintained on the standard diet prior to pairing. Males were paired with females for 14 days, allowing for insemination but also ensuring that the males were removed before females gave birth. Pups were counted, sexed, and weighed 7 days post-parturition, and were weighed again every 7 days after that until fully weaned (day 28). We weighed mothers every 7 days throughout the study, with the exception of the week following parturition. When possible, pups that died prior to weaning were sexed to determine if there was a bias in pup mortality.

**Table 1:** Composition of custom manufactured low-calcium and standard diets.

	Low-Calcium	Standard
**Composition**		
**Energy (Kcal/g)**	3.8	3.7
**Protein (% kcal from)**	18.7	19.1
**Carbohydrate (% kcal from)**	66.5	65.9
**Fat (% kcal from)**	14.8	15.0
**Calcium (% weight)**	0.10	0.85
**Phosphorus (% weight)**	0.40	0.40
**Vitamin D (IU/Kg)**	2200	2200
**Ingredients**		
**Casein (g/Kg)**	200.0	200.0
**L-Cystine (g/Kg)**	3.0	3.0
**Sucrose (g/Kg)**	334.7	330.5
**Corn Starch (g/Kg)**	270.0	255.6
**Maltodextrin (g/Kg)**	50.0	50.0
**Soybean Oil (g/Kg)**	60.0	60.0
**Cellulose (g/Kg)**	40.0	40.0
**Mineral Mix, Ca-P Deficient 79055 (g/Kg)**	18.0	18.0
**Potassium Phosphate, monobasic (g/Kg)**	11.4	11.4
**Calcium Carbonate (g/Kg)**	2.5	21.2
**Ferric Citrate (g/Kg)**	0.3	0.3
**Vitamin Mix, Teklad 40060 (g/Kg)**	10.0	10.0
**Ethoxyquin, antioxidant (g/Kg)**	0.012	0.012

Following Charnov [43], we calculated lifetime reproductive effort (LRE) of each female as the product of total number of offspring weaned and mass of offspring at independence relative to mother’s mass at first reproduction:

LRE  =  total offspring weaned × (offspring mass at independence/adult mass at first reproduction).

where offspring mass at independence was considered to be mass at day 21, and adult mass at first reproduction as the last recorded mass (within 14 days) prior to first successful fertilization. Although not completely weaned at this time, we observed pups between the ages of 21–28 days consuming solid foods, thus, we used the mass of 21 day old pups in order to more accurately represent cumulative maternal investment.

We euthanized mothers at either 85 or 100 weeks of age, approximating average lifespan of wild white-footed mice [44]. We excised the left femur, left humerus and the lumbar vertebrae to quantify skeletal condition. We measured relative bone volume, trabecular number, trabecular thickness and trabecular separation of femurs and relative bone volume of lumbar vertebrae using micro-computed tomography (MicroCT 40, Scanco Medical, Bassersdorf, Switzerland). We calculated the flexural strength of femurs and humeri on a 3-point bending fixture using monotonic axial displacement, using an 858 MiniBionix Materials Testing System with a 100 N load cell (MTS Systems Corp., Minneapolis, MN USA). The contacts of the 3-point apparatus were set at a span of 8 mm for humeri and 10 mm for femurs, and a cross head speed of 0.05 mm/sec was used to break the bone mid-shaft.

We compared maternal bone characteristics between dietary treatments using student’s t-tests. For femur characteristics, we corrected the α-value for multiple comparisons using a sequential Bonferroni for correlated variables. These values were calculated using the Qualitative Skills webpage (http://www.quantitativeskills.com). We used a general linear model (PROC GLM) to determine if maternal body mass prior to fertilization affected litter size, mean pup body mass at day 7 or mean pup mass at day 21. We used a two level nested ANOVA, with pup nested within mother, to determine if pup mass at day 7 and day 21 varied between sexes, and if maternal diet interacted with sex to affect pup body mass (PROC GLM). We used mixed models (PROC MIXED), with mother specified as a random effect and parity as a repeated variable, to test if calcium content of maternal diet, age of mother at parturition, body mass of mother prior to fertilization or parity affected the proportion of males produced per litter, litter size, or mean offspring body mass at day 7 and day 21. We selected an autoregressive covariance matrix (AR1) for these analyses based on AICC values. We used a student’s t-test to compare calculated LRE and total offspring mass produced for each mother, and to compare proportion of dead male pups between diets. We also used a chi-squared test to determine if pup mortality overall was skewed between sexes. One of the females assigned to the low-calcium diet died during the study and therefore is not included in analyses. Unless otherwise specified, statistical analyses were performed in SAS v9.2 (SAS Institute Inc., Cary, NC, USA). All means are given ± standard error.

## Results

### Maternal Bone Characteristics

Vertebral cortical bone volume was significantly lower for females that had consumed the low-calcium diet (Table 2) and there was a trend suggesting that femoral cortical bone volume could also be lower in females that consumed the low-calcium diet (Table 2) but this was not significant with Bonferroni correction. There was no significant difference between diets for any of the trabecular bone measurements of the femur (Table 2). There was no difference between diets in force required to break mothers’ femur or humerus (Table 2).

**Table 2:** Maternal bone characteristics for females consuming a low-calcium or standard diet.

	Bone	Low-Ca *X* ± SE	Standard *X* ± SE	*P*
**Cortical bone volume (BV/TV%)**	Femur	0.482±0.109	0.534±0.093	0.028‡
	Vertebrae	0.155±0.008	0.182±0.005	0.019
**Trabecular bone volume (BV/TV%)**	Femur	0.0123±0.0095	0.0177±0.0086	0.68
**Trabecular number (1/mm)**	Femur	1.41±0.121	1.37±0.118	0.86
**Trabecular thickness (mm)**	Femur	0.0350±0.0068	0.0447±0.0049	0.27
**Trabecular separation (mm)**	Femur	0.775±0.0626	0.789±0.0681	0.88
**Breaking force (N)**	Femur	14.2±0.78	15.9±0.65	0.13
	Humerus	14.3±0.48	14.1±0.84	0.86

‡ Bonferroni corrected α = 0.020.

### Reproductive Output

**Figure 1:**
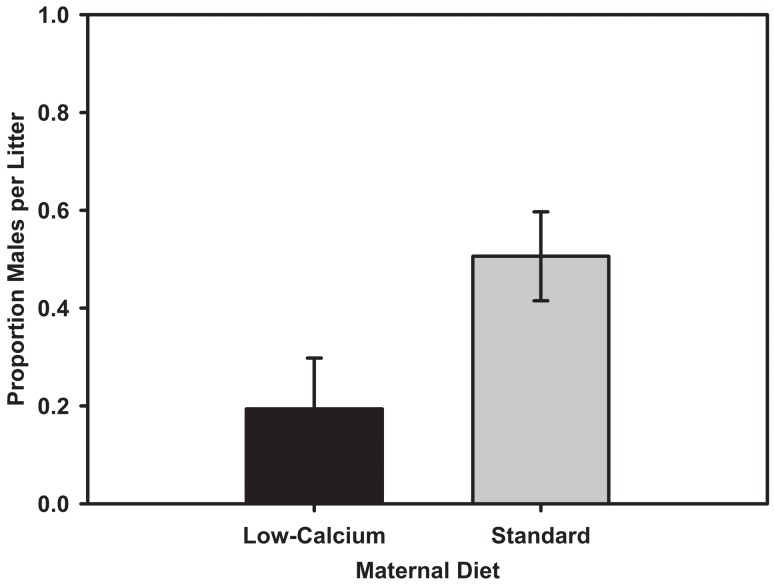
Proportion of males in litters produced by females consuming either a low-calcium or standard diet. The dashed line represents a 1∶1 sex ratio (i.e. 0.50 males).

Mothers on the low-calcium diet produced a significantly smaller proportion of males per litter on average (0.194±0.104) than females on the standard diet (0.522±0.0952; F_1,13_ = 5.47, *P* = 0.036; Fig. 1). There was no difference in body mass of male and female pups of mothers consuming the standard or low calcium diet at day 7 or 21 or pups (F≤1.60, *P*≥0.21 for all models). We were able to determine the sex of 17 pups that died prior to weaning (n_low_ = 5, n_standard_ = 12). There was no significant difference in the proportion of dead pups that were male between diets (*t*_7_ = 0.058, *P* = 0.96; low = 0.40, standard = 0.42), nor was there a significant difference between the number of male and female pups that died prior to weaning overall (χ^2^ = 0.53, *P* = 0.47).

Maternal mass prior to fertilization had no effect on litter size or mean pup mass at day 7 or day 21 (F_1,6_ = 0.84, *P* = 0.39; F_1,5_<0.001, *P* = 0.99; F_1,5_ = 0.01, *P* = 0.91; respectively), nor did diet interact with maternal mass to affect these variables (F_1,6_ = 3.74, *P* = 0.10; F_1,5_ = 0.04, *P* = 0.84; F_1,5_ = 0.21, *P* = 0.67; respectively).

Age of mother at parturition and calcium content of maternal diet each had a significant effect on litter size (Age: F_1,13_ = 5.72, *P* = 0.033; Diet: F_1,13_ = 5.38, *P* = 0.037), with litter size decreasing with age, but the interaction between age and diet was not significant (F_1,13_ = 4.24, *P* = 0.060). Mothers on the low-calcium diet produced 1–3 litters (n_1 litter_ = 4, n_2 litters_ = 1, n_3 litters_ = 1) and weaned a total of 20 pups. Mothers on the standard diet produced 1–3 litters (n_1 litter_ = 1, n_2 litters_ = 6, n_3 litters_ = 1) and weaned a total of 43 pups. Mothers on the low-calcium diet produced, on average, 2.22±0.36 pups per litter, whereas mothers consuming the standard diet produced an average of 2.69±0.28 pups per litter. Calcium content of diet, maternal age at parturition and parity had no significant effect on mean pup body mass at day 7 or day 21 (F≤2.04, *P*≥0.17 for all models, Table 3). There was no significant difference between diets for calculated LRE for individual mothers (*t*_13_ = 0.99, *P* = 0.34; Table 3). Reproductive output, mass at first pregnancy and age at parturition for each mother are shown in Table S1.

**Table 3:** Lifetime reproductive output of white-footed mice consuming a low-calcium or standard diet.

	Low-Ca*X* ± SE	Standard*X* ± SE	*P*
**Mean litter size**	2.22±0.36	2.69±0.28	0.037
**Total offspring weaned**	3.88±0.93	5.71±1.22	0.248
**Mean pup mass (g), day 7**	4.02±0.29	4.30±0.30	0.388
**Mean pup mass (g), day 21**	8.78±0.4	10.0±0. 6	0.220
**Males per litter**	0.194±0.104	0.506±0.091	0.036
**Lifetime reproductive effort** *	1.88±0.548	2.55±0.38	0.330

* Lifetime reproductive effort (LRE) based on Charnov et al. [43].

## Discussion

Given that calcium is vital for offspring skeletal development in mammals, it should follow that the availability of this resource influences reproductive performance. An individual’s fitness is determined by its own survivorship and fecundity, as well as the survivorship and fecundity of its offspring. Thus to optimize fitness, mothers must adjust their nutritive effort based on both their endogenous condition and the availability of exogenous resources. Here we have shown that nutritive allocation decisions are not only made based on the availability of energy but also with regard to available calcium.

Females subjected to a low-calcium diet throughout their lives displayed a long-term reduction in bone volume. Diminished bone volume can be interpreted as a reduction in skeletal condition, as well as a reduction in calcium reserves available for allocation to developing offspring. These females produced smaller litter sizes and bore relatively fewer male pups that female consuming a diet believed to be nutritionally complete; however pup mass at birth and weaning and lifetime reproductive effort were not affected by maternal calcium intake.

Thus, if maternal skeletal condition is used as a proxy for maternal condition, our results support the Trivers-Willard hypothesis [11]. Maternal skeletal condition at the end of the mother’s lifetime had significantly deteriorated for mothers consuming a calcium-deficient diet relative to those consuming the standard diet. Although mammals are generally capable of recouping lost bone after a reproductive event (eg. [45], [46]), the difference in skeletal condition that we observed suggests that bone loss experienced over the course of reproduction can have a sustained, long-term effect on the mother’s skeleton.

A main assumption of the Trivers-Willard hypothesis is that mothers in better condition are able to, and do, allocate more resources to their offspring. In human studies, there is some evidence that maternal calcium intake is positively related with offspring bone mineralization [47], [48]. In a concurrent study on white-footed mice, we found that mothers did indeed allocate less calcium to individual offspring when their dietary calcium intake was reduced [42]. Additionally, the mid-shaft width of femurs of pups from mothers on the standard diet was greater than that of pups produced by mothers on the low-calcium diet [42], which could affect offspring bone strength/resistance to fracture into adulthood. There is evidence from human studies that maternal diet can impact the adult skeletal phenotype of offspring (eg. [49], [50], [51], [52]); if this is also true in mice, pups receiving more calcium during development may experience long term advantages in both survival and reproduction relative to those that are calcium limited.

Mothers that consumed the low-calcium diet produced female-biased litters, whereas the sex ratio of mothers that consumed the standard diet was approximately 1∶1. Since there was no difference in pup mortality between sexes, maternal calcium intake appears to influence sex ratio prior to parturition. In this case, maternal skeletal condition represents endogenous calcium reserves that are presumably available for offspring investment, much like typical indices of condition represent energetic reserves. In mammals, the effect of maternal condition on primary sex ratio has only been consistently demonstrated when quantifying condition at conception [18], [19]. Although not directly tested, since calcium intake has both long- and short-term effects on the bone characteristics of reproductive white-footed mice, it is likely that skeletal condition at conception also differed between diets.

Interestingly, a similar reduction in litter size and number of males produced was found when laboratory mice were fed a low-fat diet, which was independent of maternal mass [53], [54]. Fat and bone are connected mechanistically through a variety of pathways (reviewed in [55]). As such, this similar effect may be indicative of a potential target for studies on the mechanisms underlying sex ratio and offspring production in mammals. The production of female-biased litters may also be a response to nutritional stress in general, as a decrease in number of males produced by rats was observed as maternal dietary salt intake increased [56].

Rosenfeld and Roberts [14] discussed several proposed mechanisms that link maternal diet and nutritional condition to skewed sex ratios in mammals. Briefly, the sperm of one sex may be differentially affected by the reproductive tract milieu in such a way as to influence motility or fertilization ability, or reproductive tract conditions may differentially affect the embryonic development of one sex over the other. Among its many functions, calcium plays a significant role in affecting sperm motility, oocyte activation, fertilization, sex differentiation of germ cells, embryo activation and differentiation of embryonic stem cells in mammals [57], [58], [59], [60], [61], [62], [63]. Therefore it is possible that any of the above mentioned functions could be mediated by maternal calcium availability.

Considerable variation in sex ratio exists within the *Peromyscus* genera, but the trend is to produce male biased litters (reviewed in [64]). However, season and maternal body mass have been associated with skewed sex ratios, with male biased litters produced in spring, when females are heavier, and female biased litters produced in fall, when mothers have reduced body mass [65] which likely reflects seasonal fluctuations in food abundance or a depletion of somatic resources over the reproductive season. In our study, females on the low-calcium diet were heavier than those on the standard diet. This may be due to females attempting to compensate for low calcium content of their food by consuming more food overall; however, given the disparate calcium content between the two diets, that the calcium intake of females on the low-calcium diet did not approach that of females on the standard diet.

Sources of supplemental calcium may not be consistently available to wild rodents and other vertebrates that consume a low calcium diet. Availability of calcium may vary temporally, as in seasonal access to shed antlers, or geographically, as soils, and thus invertebrate prey, may either be low in calcium due to natural variation or anthropogenic disturbance [66], [67]. Thus, we predict that wild white-footed mice will also produce smaller, female-biased litters when their access to calcium is limited. In this investigation, female white-footed mice adjusted their reproductive strategy in response to low calcium intake in a manner that both mimicked empirical evidence and followed theoretical predictions for low energy availability, suggesting that a mother’s skeletal condition, like available fat stores, should be considered in models of reproductive trade-offs.

## Supporting Information

Table S1 **Reproductive output and pup morphological data for mothers consuming either a low-calcium or standard diet.**
(DOCX)
