# Supplementary material for: Calcium Availability Influences Litter Size and Sex Ratio in White-Footed Mice (Peromyscus leucopus)
Source: PLoS One. 2012 Aug 1;7(8):e41402. doi: 10.1371/journal.pone.0041402 (PMC3409861; doi:10.1371/journal.pone.0041402)
Supplement: Table S1 — Reproductive output and pup morphological data for mothers consuming either a low-calcium or standard diet. (DOCX) [file pone.0041402.s001.docx]

**Table S1.** Reproductive output and pup morphological data for mothers consuming either a low-calcium or standard diet.

| **Mother id** | **Mass at 1^st^ pregnancy (g)** | **Age at parturition (weeks)** | **Litter size** | **Number of male pups** | **Mean pup mass day 7 (g)** | **Mean pup tail length day 7 (mm)** | **Mean pup mass day 21 (g)** | **Mean pup tail length day 21(mm)** | **Total litter mass day 21 (g)** |
| --- | --- | --- | --- | --- | --- | --- | --- | --- | --- |
|  |  |  |  |  |  |  |  |  |  |
| *Low-calcium diet* | | | | | | | | | |
| 29 | 19.50 | 44 | 3 | 1 | 5.78 | 37.3 | 9.60 | 50.0 | 28.80 |
| 39 | 21.93 | 19 | 1 | 0 | 4.47 | 24.50 | 9.67 | 62.1 | 9.67 |
|  |  | 33 | 4 | 3 | 4.02 | 19.9 | 9.52 | 49.7 | 38.06 |
|  |  | 66 | 3 | 2 | 4.39 | 22.5 | 8.97 | 53.5 | 26.90 |
| 72 | 18.80 | 53 | 3 | 0 | 3.96 | 21.8 | 8.06 | 47.0 | 24.19 |
| 77 | 21.51 | 31 | 2 | 0 | 3.48 | 17.1 | 8.02 | 42.8 | 16.03 |
| 86 | 19.97 | 29 | 2 | 0 | 3.75 | 22.0 | 9.58 | 56.1 | 19.16 |
| 94b | 20.00 | 57 | 1 | 0 | 3.78 | 20.2 | 9.60 | 55.4 | 9.60 |
|  |  | 75 | 1 | 0 | 2.53 | 18.2 | 6.01 | 43.6 | 6.01 |
| *Standard calcium diet* | | | | | | | | | |
| 2 | 18.60 | 64 | 2 | 0 | 5.23 | 24.4 | 10.35 | 58.2 | 20.70 |
|  |  | 78 | 1 | 1 | 5.02 | 24.3 | 10.39 | 55.8 | 10.39 |
| 30 | 18.89 | 38 | 3 | 3 | 5.06 | 21.5 | 11.02 | 48.4 | 33.07 |
|  |  | 69 | 3 | 2 | 4.05 | 21.2 | 9.10 | 55.2 | 27.3 |
| 75 | 20.73 | 59 | 3 | 2 | 5.35 | 20.3 | 12.27 | 54.2 | 36.82 |
|  |  | 77 | 1 | 0 | 4.65 | 26.4 | 10.69 | 65.3 | 10.69 |
| 81 | 17.90 | 48 | 3 | 0 | 4.26 | 23.6 | 8.61 | 56.1 | 25.84 |
|  |  | 59 | 2 | 1 | 5.28 | 24.1 | 9.82 | 53.5 | 19.64 |
| 92 | 18.65 | 47 | 3 | 1 | 4.05 | 21.7 | 8.75 | 49.5 | 26.26 |
|  |  | 60 | 3 | 1 | 3.05 | 17.4 | 7.51 | 48.0 | 22.53 |
| 3b | 21.84 | 24 | 4 | 2 | 4.74 | 22.8 | 11.30 | 54.1 | 45.18 |
|  |  | 52 | 1 | 0 | 5.84 | 23.9 | 12.09 | 57.7 | 12.09 |
|  |  | 70 | 3 | 3 | 5.37 | 21.2 | 15.48 | 58.8 | 46.43 |
| 74a | 16.20 | 74 | 2 | 2 | 4.18 | 24.4 | 9.18 | 53.1 | 18.35 |
| 83b | 20.44 | 31 | 5 | 3 | 3.05 | 17.9 | 5.95 | 38.3 | 29.73 |
|  |  | 48 | 4 | 3 | 3.60 | 21.8 | 8.10 | 48.2 | 32.39 |
